# Identification of potent HDAC 2 inhibitors using E-pharmacophore modelling, structure-based virtual screening and molecular dynamic simulation

**DOI:** 10.1007/s00894-022-05103-0

**Published:** 2022-04-13

**Authors:** Padmini Pai, Avinash Kumar, Manasa Gangadhar Shetty, Suvarna Ganesh Kini, Manoj Bhat Krishna, Kapaettu Satyamoorthy, Kampa Sundara Babitha

**Affiliations:** 1 Department of Biophysics, Manipal School of Life Sciences, Manipal Academy of Higher Education, Manipal, Karnataka 576104 India; 2 Department of Pharmaceutical Chemistry, Manipal College of Pharmaceutical Sciences, Manipal Academy of Higher Education, Manipal, Karnataka 576104 India; 3 Department of Bioinformatics, Manipal School of Life Sciences, Manipal Academy of Higher Education, Manipal, Karnataka 576104 India; 4 Department of Cell and Molecular Biology, Manipal School of Life Sciences, Manipal Academy of Higher Education, Manipal, Karnataka 576104 India

**Keywords:** HDAC 2 inhibitors, Structure-based virtual screening, E-pharmacophore model, Selective inhibition, Molecular dynamics

## Abstract

Histone deacetylase 2 (HDAC 2) of class I HDACs plays a major role in embryonic and neural developments. However, HDAC 2 overexpression triggers cell proliferation by diverse mechanisms in cancer. Over the decades, many pan and class-specific inhibitors of HDAC were discovered. Limitations such as toxicity and differential cell localization of each isoform led researchers to hypothesize that isoform selective inhibitors may be relevant to bring about desired effects. In this study, we have employed the PHASE module to develop an e-pharmacophore model and virtually screened four focused libraries of around 300,000 compounds to identify isoform selective HDAC 2 inhibitors. The compounds with phase fitness score greater than or equal to 2.4 were subjected to structure-based virtual screening with HDAC 2. Ten molecules with docking score greater than  -12 kcal/mol were chosen for selectivity study, QikProp module (ADME prediction) and dG/bind energy identification. Compound 1A with the best dock score of  -13.3 kcal/mol and compound 1I with highest free binding energy,  -70.93 kcal/mol, were selected for molecular dynamic simulation studies (40 ns simulation). The results indicated that compound 1I may be a potent and selective HDAC 2 inhibitor. Further, *in vitro* and *in vivo* studies are necessary to validate the potency of selected lead molecule and its derivatives.

**Graphical abstract:**

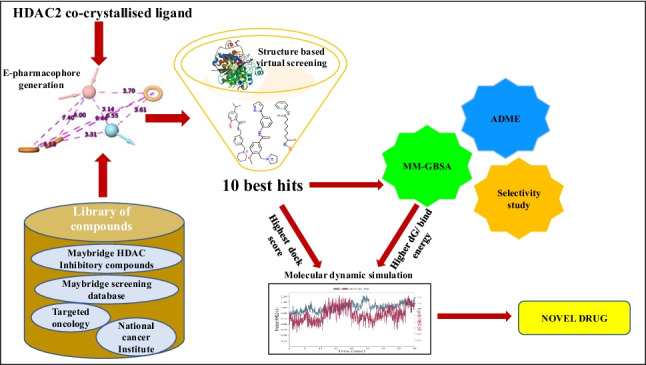

**Supplementary Information:**

The online version contains supplementary material available at 10.1007/s00894-022-05103-0.

## Introduction

Chromatin comprises the repeating units of nucleosomes and facilitates to embed the DNA in the core. Chromatin consists of H2A, H2B, H3 and H4 histone proteins (two molecules of each histone proteins form an octamer) crossing roughly 147 bp of DNA has been subjected to intense research [1]. For the past few years, there has been significant progress in our knowledge on the different types of histone modifications such as phosphorylation, methylation, acetylation and ubiquitination. Acetylation and deacetylation modifications of lysine present in H3 and H4 are facilitated by the activities of histone acetyltransferases (HATs) and histone deacetylases (HDACs) respectively. The activity of HATs is associated with a relaxed chromatin structure and transcription promotion, while HDACs activity promotes compact chromatin structure and downregulates the transcription. Till now, eighteen diverse types of HDACs are discovered and classified into four groups: class I (HDAC 1, 2, 3 and 8), class II (HDAC 4, 5, 6, 7, 9 and 10), class III (sirtuins: NAD-dependent enzymes) and class IV (HDAC 11). These isoforms are recognised as potential therapeutic targets due to their significant role in different diseases such as cancer, inflammation, neurological and lung disorders [2, 3].

Histone deacetylase inhibitors (HDACIs) can induce cell cycle arrest and apoptosis. Hence, they are attracted considerable interest as therapeutically potent scaffolds [4]. Recent data suggest that HDACIs enhance cognitive ability and repair neurodegenerative impairment thereby helps to re-establish long-term memory [5]. The drugs that have been discovered to date are pan inhibitors that target all the HDAC isoforms or class selective inhibitors. Few pan HDAC inhibitors such as suberoylanilide hydroxamic acid (SAHA), belinostat and panobinostat have been approved to treat cutaneous T-cell lymphoma, peripheral T-cell lymphoma and multiple myeloma, respectively. Some of the HDAC class-specific inhibitors reported are nanatinostat (class I) and rocilinostat (class II) [6]. These inhibitors affect the global acetylation and deacetylation process which in turn alters large number of genes. Even though pan inhibitors are potent therapeutics, they can be toxic due to simultaneous inhibition of several isoforms. Therefore, second-generation HDAC inhibitors are focused on isoform-selective compounds and can serve as potent drug molecules for several diseases [7].

Current work focuses on HDAC 2, which belongs to class I HDACs. HDAC 2 plays a vital role in embryonic, neural development and cardiac functions [8]. However, literature survey has shown that HDAC 2 is overexpressed leading to proliferation of oral, breast and colon cancers [9–11]. Aberrant HDAC 2 influences the expression of tumour suppressor genes such as p21 (WAF1/Cip1) and p53 [12, 13]. HDAC 2 is downregulated by microRNAs such as miR-200 and miR-145 and promotes apoptosis [14, 15]. Moreover, selective HDAC 1/HDAC 2 inhibition induces neuroblastoma differentiation and reduces cell viability [16]. Furthermore, HDAC 2 modulates synaptic plasticity and long-lasting changes of neural circuits may negatively regulate the processes of learning and memory [17]. Aberration in HDAC 2 affects several pathways such as NF-kB and STAT1 signalling [18, 19]. Even though HDAC 2 plays a crucial role in various diseases, till date no compounds have been approved as an isoform selective HDAC 2 inhibitor. Therefore, recently many researchers have focused on the development of isoform selective HDAC 2 inhibitors [20].

Computational methods help to reduce drug development cost by screening large databases. It analyses the interaction between the ligand and the protein in a biological environment. Many novel lead molecules were discovered using virtual screening [21–23]. Despite being a promising approach in drug discovery, very few drugs based on virtual screening have entered into clinical studies and these include PRX-03140 (phase IIB) and PRX-08066 (phase IIA) to treat Alzheimer’s disease and pulmonary hypertension, respectively [24]. Similar approaches were used to identify potent HDAC inhibitors. A comparative structure and ligand-based *in silico* study was carried out to explore the structural requirements of isoform selective HDACIs [25]. In another study, non-hydroxamic acid based HDAC inhibitors were recognised and the lead compound obtained was evaluated for its HDAC inhibitory and anticancer activity *in vitro* [26]. HDAC 8 isoform selective inhibitors were identified by in silico study, using 167,000 molecules and the three best hits were filtered based on factors such as rule of five, presence of zinc-binding groups (ZBG), binding pattern and pharmacophore models. The hits were then subjected to *in vitro* enzyme inhibition assays [27]. Similarly, in another study, potent HDAC 8 inhibitors were identified from a library of 4.3 × 10^6^ molecules [22]. Novel HDAC 6 selective inhibitors were discovered from library of 330,000 compounds and tested for HDAC 6 inhibition and cytotoxicity [28]. HDAC 2 selective inhibitors were identified using quantum polarised ligand docking, pharmacophore generations and binding free energy calculation [29]. In another study, 3D QSAR pharmacophore generation and structure-based virtual screening were used to recognise HDAC 2 selective inhibitors [21].

In the present study, we have combined e-pharmacophore, structure-based virtual screening, free binding energy calculation and molecular dynamic simulation to discover novel HDAC 2 selective inhibitors.

## Materials and methods

### Protein preparation for docking studies

All the studies were performed with Maestro version 11.4 (Schrodinger Inc.) The crystal structures of different isoforms of HDACs were retrieved from the Protein Data Bank (Table 1).

**Table 1:** Classification of HDAC isoforms and their PDB IDs

Classification	HDAC Isoform	PDB ID
Class I	HDAC 1	4BKX
	HDAC 2	4LY1
	HDAC 3	4A69
	HDAC 8	1T69
Class IIA	HDAC 4	2VQJ
	HDAC 5	–^a^
	HDAC 7	3ZNR
	HDAC 9	–^a^
Class IIB	HDAC 6	3PHD
	HDAC 10	6UII
Class IV	HDAC 11	–^a^

^a^Protein modelling using SWISS model

These structures were subjected to protein preparation wizard (PPW), missing hydrogens were added and the metal ionisation state was corrected to maintain the formal charge and force field [30]. Protein preparation wizard has three-step workflow as follows: (1) pre-processing, (2) review and modify and (3) minimise. In the pre-processing steps, the PPW tool automatically identifies any problem with the imported protein structure, like missing hydrogen atoms, missing side chains and missing loops, and rectifies them as per its inbuilt algorithm. PPW can assign bond orders, create zero-order bonds to metals and create disulphide bonds. PPW employs integrated prime functionality to fill missing side chains or loops. In the second step, it can generate het states using Epik at any specified pH. For minimization, OPLS3e force field was used [31]. The crystal structure of HDAC 5 (PDB ID: 5UWI) did not had Zn^2+^ ion and crystal structures of HDAC 9 and 11 were unavailable in the Protein Data Bank. Therefore, we have obtained 3D protein model for HDAC 5 (Q9UQL6), HDAC 9 (Q9UKV0) and HDAC 11 (Q96DB2) using SWISS-MODEL [32] (https://swissmodel.expasy.org/).

### Database selection and ligand preparation

We have utilised four different databases: (a) targeted oncology from Asinex (6,728 compounds), (b) National Institute of Health (NIH) from National Cancer Institute (NCI) (237,000 compounds), (c) screened compounds from Maybridge (4,107 compounds) and (d) HDAC inhibitors from Maybridge (53,352 compounds). These compounds were subjected to Ligprep module (version 44,011) to generate 3D structures with low energy retaining the originality of chirality and ionisation. Force field applied was OPLS 2003e to produce minimal energy structures with corrected chirality [30]. SAHA and MS-275 are known pan and class I HDAC inhibitors, respectively and considered as positive controls.

### E-pharmacophore modelling and virtual screening

E-pharmacophore-based virtual screening combines structure and ligand-based approaches and carried out by PHASE module maestro Schrodinger, which is used to screen the compounds based on e-pharmacophore generated [33, 34]. In the current study, we generated e-pharmacophore by using co-crystallised ligand 4-(acetylamino)-N-[2-amino-5-(thiophen-2-yl)-phenyl]-benzamide (selective for HDAC 1 and HDAC 2 isoforms) with the HDAC 2 protein [35]. The compounds which fulfil the hypothesis were selected for the further study.

### Structure-based virtual screening

Grid box of HDAC 2, 3, 8, 4 and 7 proteins was made at the site of a co-crystallised ligand. SiteMap module comprises of an algorithm to locate binding sites and can be used to setup grid boxes [36, 37]. Active sites of HDAC 1, 6 and 10 were identified using the SiteMap module. Since FDA-approved HDAC inhibitors chelate Zn^2+^ ion to inhibit its activity, we have considered amino acids surrounding Zn^2+^ ion to create the grid box of modelled proteins. HDAC 5 protein grid box was generated including PRO 22, HID 24, ASN 216, PHE 217, PHE 218, ASP 281, PRO 289, LEU 290, GLY 291, GLY 321, GLY 322 and HID 323 residues. HDAC 9 grid box was made by including THR 22, THR 23, HID 24, PRO 25, GLU 26, ASP 284, PRO 292, LEU 293, GLY 294, GLY 324, GLY 325 and HID 326 residues. HDAC 11 protein grid box was formed by including GLY 125, GLY 126, GLY 127, GLY 136, CYS 140, ILE 195, TRP 196, ASP 248, GLY 256, GLY 289 and GLY 290 residues. Glide offers rapid vs precision options ranging from high-throughput virtual screening (HTVS) - capable of screening large compound libraries, standard precision (SP) - up to hundreds of compounds with high precision and extra precision (XP) - highly accurate models eliminating false positives [38–40].

### Free binding energy calculation (MM-GBSA)

Docking of identified ligands demonstrates efficient binding to the active site of protein. However, the protein–ligand association should continue in the same state to promote any potential biological response. These responses mainly depend upon the free binding energy. Therefore, ten best hits are subjected to prime module to determine the free binding energy.

### ADME prediction

ADME (absorption, distribution, metabolism and excretion) prediction is discovered to reduce the late stage failures in the drug development process. We have used QikProp version 5.4 of Maestro module to predict the ADME properties of best ten hits. Properties such as molecular weight, donor HB (number of H-bond donors), acceptor HB (number of H-bond acceptors), QPlogPO/w (Octonol/water partition coefficient), QPlogS (predicted aqueous solubility), QPPCaco (Caco-2 permeability), QPlogBB (blood/brain partition coefficient), HOA (qualitative human oral absorption value ranges from 1 for low, 2 for medium and 3 for high), PHOA (percentage of human absorption), QPlogKhsa (binding to human serum albumin), ROF (rule of five), PSA (polar surface area), Metab (number of likely metabolic reactions) and ROT (rule of three) were measured.

### Molecular dynamics simulation

Molecular dynamics (MD) simulation is done to overcome the disadvantages of molecular docking studies. It provides flexible receptor-ligand interaction by solvating the system. Compounds IA and 1I, with high dock score and with high dG/binding score, respectively, were subjected to molecular dynamics simulation for the better understanding of the stability of protein–ligand interactions. MD simulation studies were performed using Desmond module of Schrodinger. It has a three-step workflow where system builder was the first step. In this step, ligand-protein complex was solvated using simple point charge (SPC) solvent model in an orthorhombic box shape. SPC is a three-site solvent model widely used in MD simulations studies for small molecule-protein complexes. SPC assumes an ideal tetrahedral shape (HOH angle of 109.47 °) instead of the observed angle of 104.5 °. Second step was the minimisation of the solvated ligand-protein complex using steepest descent (SD) method with maximum iterations fixed in 2000 and convergence threshold at 1 kcal/mol/Å. Slow relaxation protocol was followed for the minimised complex, and it was calibrated at a temperature and pressure of 300 K and 1 bar, respectively. Nose–Hoover method was used as thermostat and Martina–Tobias–Klein method was used as barostat. The last step was simulating this minimised complex for 40 ns. A frame was captured every 40 ps and thus a total of 1000 frames were generated. RMSD plots, RMSF plots, ligand interaction diagrams, histogram plots etc. were generated to analyse the results of MD simulation studies [41].

## Results and discussion

### E-pharmacophore modelling and virtual screening

**Fig. 1:**
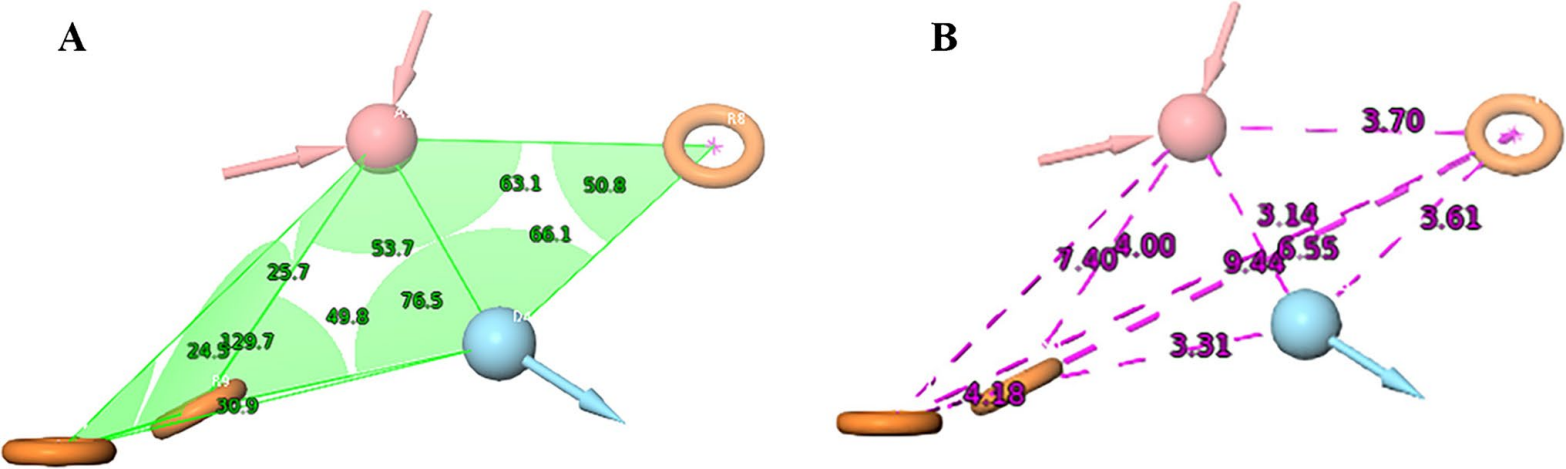
A five-feature e-pharmacophore (RRRAD) model generated using PHASE module for selective HDAC 2 inhibitor illustrating hydrogen bond acceptor (pink sphere), hydrogen bond donor (sky blue sphere) and aromatic ring (orange rings). **A** green area indicated inter-site angle between features and **B** purple lines indicate inter-site distance between features

The ligand 4-(acetylamino)-N-[2-amino-5-(thiophen-2-yl)-phenyl]-benzamide, which is co-crystallised with HDAC 2, is used as a reference for the e-pharmacophore generation [35]. E-pharmacophore hypothesis consists of three aromatic rings (R7, R8 and R9), one H-bond acceptor (A2) and one H-bond donor (D4) (Fig. 1). This hypothesis is used to form a basic skeleton of compounds with specific angle and distance which is likely to bind to HDAC 2.

Formed e-pharmacophore is used to screen the libraries of compounds and subjected to Ligprep module, which generated 17,054; 443,161; 8,210 and 83,797 compounds from targeted oncology database, National Cancer Institute database, HDAC inhibitory compounds collection and screening collection database from Maybridge, respectively. Compounds matching with minimum of three criteria in the hypothesis were selected. Among these, compounds with phase fitness score more than or equal to 2.4 were chosen for structure-based virtual screening. Targeted oncology produced 6,124 compounds. However, all the compounds had phase fitness score less than 2.4. Hence, 17,054 compounds generated in Ligprep were directly subjected to structure-based virtual screening. Similarly, 7 out of 38,578 and 79 out of 4,127 compounds were selected from NCI and HDAC inhibitory compounds collection database from Maybridge. All the 6,088 compounds obtained from Maybridge screening database had phase fitness score less than 2.4 and hence not considered for further analysis.

### Structure-based virtual screening and MM-GBSA

**Fig. 2:**
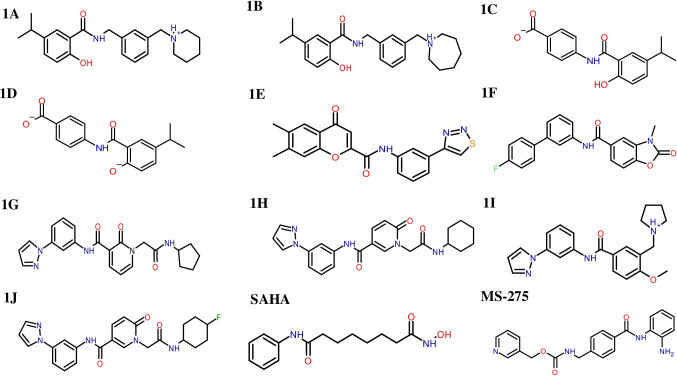
Chemical structures of best ten hits through structure-based virtual screening and control drugs SAHA and MS-275

17,054 compounds selected from targeted oncology database from Asinex were subjected to HTVS mode of docking. One hundred forty two compounds with dock score more than or equal to -9 kcal/mol were chosen for SP docking. Fifty compounds with SP dock score more than or equal to -10 kcal/mol were subjected to XP docking. Finally, 4 compounds (1A to 1D) with XP dock score above -12 kcal/mol were selected. Similarly, 86 compounds from NCI and HDAC inhibitors from Maybridge were subjected to XP mode of docking and 6 compounds (1E to 1 J) with XP dock score more than -12 kcal/mol were selected. Chemical structures of best 10 hits (Fig. 2) are shown below.

To check the selectivity, ligands were docked with other HDAC isoforms. XP docking scores of the selected ligands with HDAC 2 were greater than -12 and greater than the positive controls, SAHA and MS-275 (-11.6 kcal/mol) (Table 2). Compounds showed higher docking scores towards HDAC 2, compared to all other HDAC isoforms. The XP dock score with HDAC isoforms ranges from -2.0 to -13.3 kcal/mol. To validate the docking study, the co-crystallised ligand of HDAC 2 in the PDB ID is redocked and RMSD value was found to be 0.25 Å. The ligand poses and the interacted amino acids are compared between X-ray crystallography and redocked ligand–protein complex (Figs. S1 and S2).

**Table 2:** XP dock scores of best ten hits with all the HDAC isoforms and dG/binding energy with HDAC 2

Ligand	Docking score (kcal mol^−1^)											Prime MM-GBSA (dG bind kcal mol^−1^)
	HDAC1	HDAC2	HDAC3	HDAC8	HDAC4	HDAC5	HDAC7	HDAC9	HDAC6	HDAC10	HDAC11	
1A	− 3.2	− 13.3	− 6.0	− 11.9	− 8.5	− 4.0	− 9.0	− 3.7	− 3.1	− 10.1	NA	− 55.3
1B	− 2.7	− 12.6	− 6.0	− 10.0	− 9.9	− 4.2	− 8.7	− 3.8	− 4.0	− 7.2	NA	− 58.7
1C	− 2.5	− 12.3	− 5.4	− 10.8	− 6.9	− 3.3	NA	− 3.3	NA	NA	− 4.2	− 42.6
1D	− 2.4	− 12.2	− 4.9	− 10.0	− 2.9	− 2.0	NA	− 3.5	− 3.9	− 4.0	− 3.6	25.3
1E	− 1.7	− 12.8	− 4.1	− 8.0	− 6.9	− 2.7	NA	− 3.4	NA	− 4.7	− 3.1	− 65.0
1F	− 2.4	− 12.4	− 4.7	− 5.7	− 5.4	− 2.8	NA	− 2.9	− 4.1	− 5.7	− 3.1	− 57.3
1G	− 3.0	− 12.4	− 4.6	− 8.6	− 7.3	− 4.0	NA	− 3.6	NA	NA	NA	− 62.2
1H	− 2.6	− 12.3	− 4.9	− 7.5	− 8.0	− 2.5	NA	− 2.7	NA	NA	NA	− 64.5
1I	− 2.6	− 12.1	− 5.7	− 4.9	− 6.5	− 5.5	NA	− 5.8	NA	− 5.8	NA	− 70.9
1 J	− 2.2	− 12.1	− 5.2	− 7.5	− 8.1	− 3.5	NA	− 3.0	NA	NA	NA	− 70.3
SAHA	− 2.9	− 11.6	− 1.7	− 9.9	− 8.0	− 4.8	− 7.9	− 2.8	− 2.6	− 9.1	− 8.2	− 48.7
MS-275	− 2.9	− 11.6	− 6.9	− 11.3	− 7.7	− 3.3	− 8.6	− 3.1	− 4.5	− 4.6	NA	− 50.0

*NA*, no pose viewer file generated

**Fig. 3:**
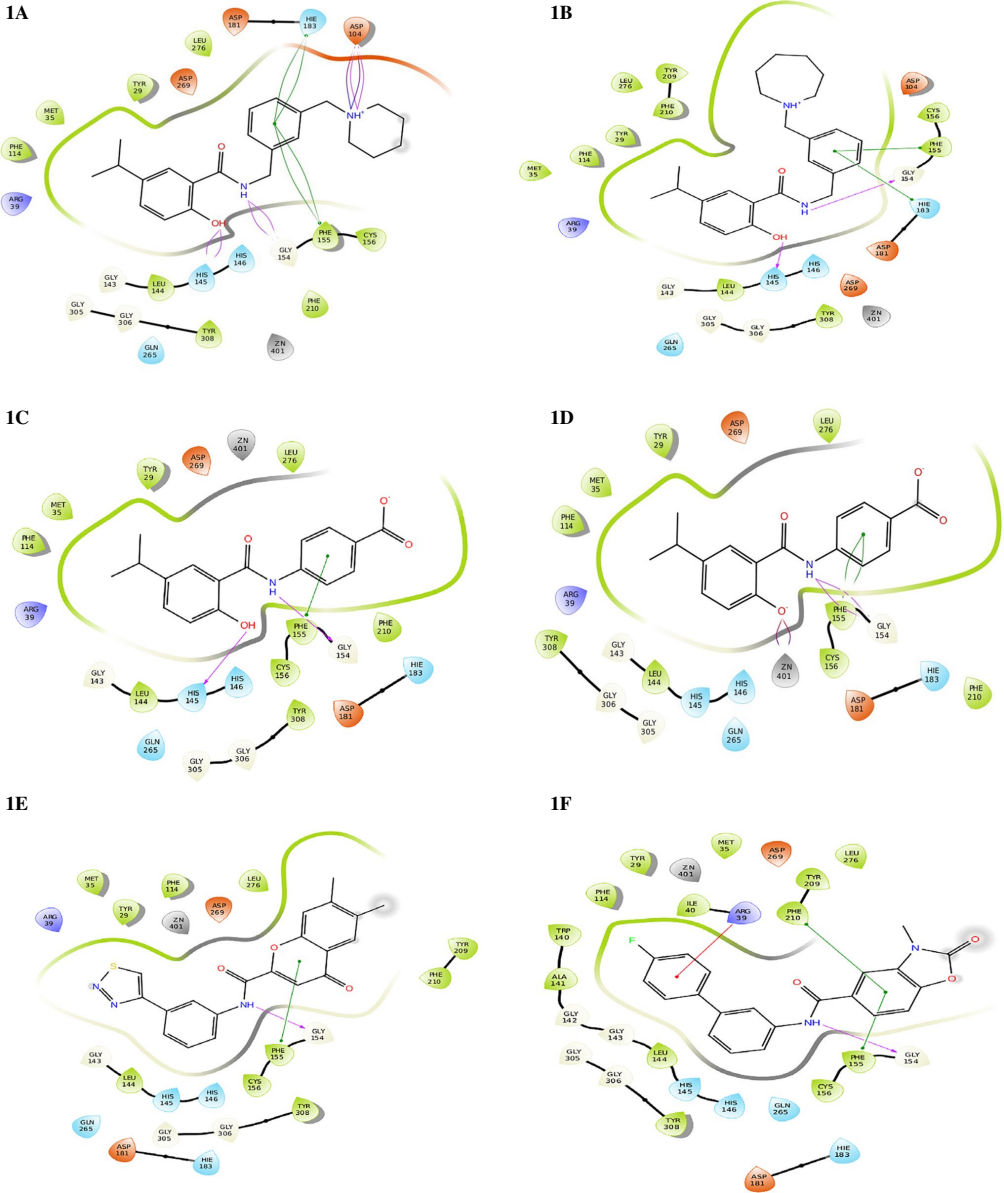

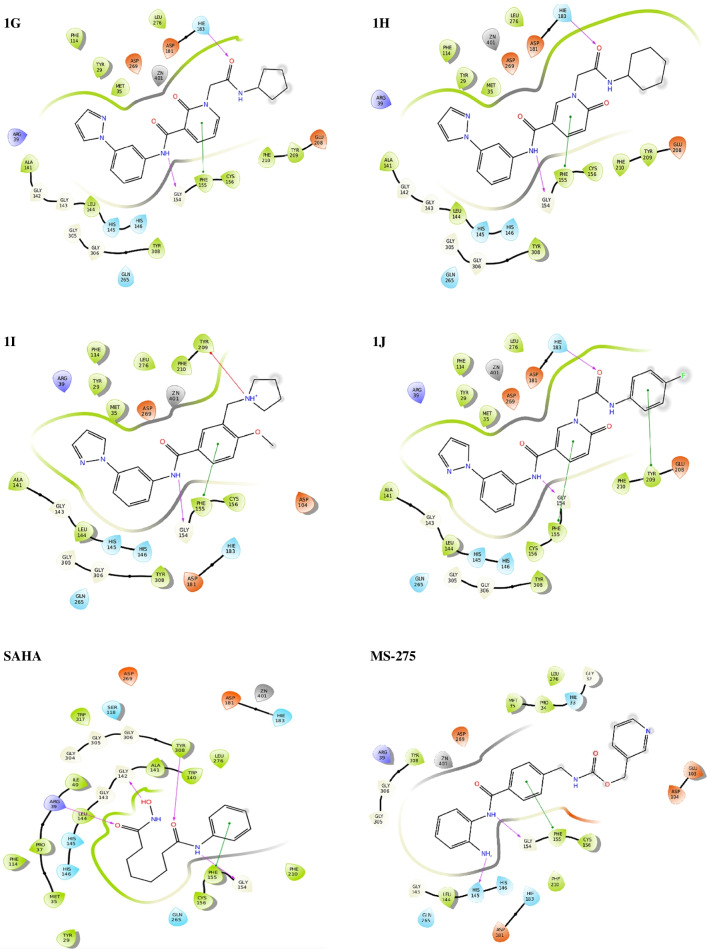
2D diagram depicting HDAC 2-ligand interaction of best 10 hits and known HDAC inhibitors SAHA and MS-275. Key amino acids and their binding interaction were identified

Compound 1A showed maximum XP dock score with the value of -13.3 kcal/mol, followed by compounds 1E and 1B with the values of  -12.8 kcal/mol^1^ and -12.6 kcal/mol, respectively. Among the ten hits, compound 1I showed better isoform selectivity towards HDAC 2 with dock score of -12.1 kcal/mol and dock score less than  -7 kcal/mol for other isoforms. Best 10 hits were subjected to MM-GBSA analysis with HDAC 2 and free binding energy was determined (Table 2). Compound 1I showed better dG/binding energy of  -70.9 kcal/mol, followed by compound 1J with the value of  -70.3 kcal/mol^−1^. 2D interaction of 10 best hits with HDAC 2 was compared to SAHA and MS-275 (Fig. 3).

Compound 1A showed hydrophobic interaction with TYR 29, MET 35, PHE 114, LEU 144, PHE 155, CYS 156, PHE 210, LEU 276, TYR 308; hydrogen bond interaction with ASP 104, HIS 145 and GLY 154; π-π stacking with PHE 155 and HIE 183; water interaction with GLY 143, GLY 154, GLY 305 and GLY 306 and polar interaction with HIS 145, HIS 146, HIE 183 and GLN 265.

Compound 1I demonstrated hydrophobic interaction with TYR 29, MET 35, PHE 114, ALA 141, LEU 144, PHE 155, CYS 156, TYR 209, PHE 210, LEU 276 and TYR 308; hydrogen bond interaction with GLY 154; π-π stacking with PHE 155; π-π cation interaction with TYR 209; water interaction with GLY 143, GLY 154, GLY 305 and GLY 306 and polar interaction with HIS 145, HIS 146, HIE 183 and GLN 265.

SAHA showed hydrophobic interaction with TYR 29, MET 35, PRO 37, ILE 40, PHE 114, TRP 140, ALA 141, PHE 155, CYS 156, PHE 210, LEU 276, TYR 308 and TRP 317; H-bond interaction with ARG 39, GLY 142, GLY 154 and TYR 308; π-π stacking with PHE 155; water interaction with GLY 142, GLY 143, GLY 154, GLY 304, GLY 305 and GLY 306 and polar interaction with SER 118, HIS 145, HIS 146, HIE 183 and GLN 265.

Similarly, MS-275 has shown hydrophobic interactions with PRO 34, MET 35, LEU 144, PHE 155, CYS 156, PHE 210, LEU 276 and TYR 308; H-bond interaction with HIS 145 and GLY 154; π-π stacking with PHE 155; water interaction with GLY 32, GLY 154, GLY 305 and GLY 306 and polar interaction with HIE 33, HIS 145, HIS 146, HIE 183 and GLN 265. Important interactions of selected and control compounds SAHA and MS-275 are mentioned in Table 3.

**Table 3:** Summary of important interactions of selected and control compounds SAHA and MS-275 with HDAC 2

Ligand	Hydrophobic interactions	H-bond interactions	Polar interaction	Any other interactions
1A	TYR 29, MET 35, PHE 114. LEU 144, PHE 155, CYS 156, PHE 210, LEU 276, TYR 308	ASP 104, HIS 145, GLY 154	HIS 145, HIS 146, HIE 183, GLN 265	Positive: ARG 39 Negative: ASP 181, ASP 104, ASP 269 Pi-pi stacking: PHE 155, HIE 183
1B	TYR 29, MET 35, PHE 114, LEU 144, PHE 155, CYS 156, TYR 209, PHE 210, LEU 276, TYR 308	HIS 145, GLY 154	HIS 145, HIS 146, GLN 265, HIE 183	Positive: ARG 39 Negative: ASP 104, ASP 181, ASP 269 Pi-pi stacking: PHE 155, HIE 183
1C	TYR 29, MET 35, PHE 114, LEU 144, PHE 155, CYS 156, PHE 210, LEU 276, TYR 308	HIS 145, GLY 154	HIS 145, HIS 146, HIE 183, GLN 265	Positive: ARG 39 Negative: ASP 181, ASP 269 Pi-pi stacking: PHE 155
1D	TYR 29, MET 35, PHE 114, LEU 144, PHE 155, CYS 156, PHE 210, LEU 276, TYR 308	GLY 154	HIS 145, HIS 146, HIE 183, GLN 265	Positive: ARG 39 Negative: ASP 181, ASP 269 Pi-pi stacking: PHE 155
1E	TYR 29, MET 35, LEU 144, PHE 155, CYS 156, TYR 209, PHE 210, LEU 276, TYR 308	GLY 154	HIS 145, HIS 146. HIE 183, GLN 265	Positive: ARG 39 Negative: ASP 181, ASP 269 Pi-pi stacking: PHE 155
1F	TYR 2, MET 35, ILE 40, PHE 114, TRP 140, ALA 141, LEU 144, PHE 155, CYS 156, TYR 209, PHE 210, LEU 276, TYR 308	GLY 154	HIS 145, HIS 146, HIE 183, GLN 265	Positive: ARG 39 Negative: ASP 181, ASP 269 Pi-pi stacking: PHE 155, PHE 210
1G	TYR 29, MET 35, PHE 114, ALA 141, LEU 144, PHE 155, CYS 156, TYR 209, PHE 210, LEU 276, TYR 308	GLY 154, HIE 183	HIS 145, HIS 146, HIE 183, GLN 265	Positive: ARG 39 Negative: GLU 208, ASP 181, ASP 269 Pi-pi stacking: PHE 155
1H	TYR 29, MET 35, PHE 114, ALA 141, LEU 144, PHE 155, CYS 156, TYR 209, PHE 210, LEU 276, TYR 308	GLY 154, HIE 183	HIS 145, HIS 146, HIE 183, GLN 265	Positive: ARG 39 Negative: GLU 208, ASP 269, ASP 181 Pi-pi stacking: PHE 155
1I	TYR 29, MET 35, PHE 114, ALA 141, LEU 144, PHE 155, CYS 156, TYR 209, PHE 210, LEU 276, TYR 308	GLY 154	HIS 145, HIS 146, HIE 183, GLN 265	Positive: ARG 39 Negative: ASP 104, ASP 181, ASP 269 Pi-pi stacking: PHE 155
1 J	TYR 29, MET 35, PHE 114, ALA 141, LEU 144, PHE 155, CYS 156, TYR 209, PHE 210, LEU 276, TYR 308	GLY 154, HIE 183	HIS 145, HIS 146, HIE 183, GLN 265	Positive: ARG 39 Negative: ASP 181, GLU 208, ASP 269 Pi-pi stacking: PHE 155, TYR 209
SAHA	TYR 29, MET 35, PRO 37, ILE 40, PHE 114, TRP 140, ALA 141, PHE 155, CYS 156, PHE 210, LEU 276, TYR 308, TRP317	ARG 39, GLY 142, GLY 154, TYR 308	SER 118, HIS 145, HIS 146, HIE 183, GLN 265	Positive: ARG 39 Negative: ASP 181, ASP 269 Pi-pi stacking: PHE 155
MS-275	PRO 34, MET 35, LEU 144, PHE 155, CYS 156, PHE 210, LEU 276, TYR 308	HIS 145, GLY 154	HIE 33, HIS 145, HIS 146, HIE 183, GLN 265	Positive: ARG 39 Negative: GLU 103, ASP 104, ASP 181, ASP 269 Pi-pi stacking: PHE 155

### ADME prediction

ADME prediction was carried out by QikProp module and showed that the compounds selected could be promising HDAC 2 inhibitors (Table 4). All the compounds had recommended values for molecular weight (Mol. wt), hydrogen bond donor ability (donorHB), hydrogen bond acceptor ability (AccptHB), water/gas partition coefficient (QPlogPo/w) and aqueous solubility (QPlogS). Caco-2 cell permeability is predicted by QPPCaco and ranges from 41.69 to 851.00. The compounds 1F and 1I have shown great permeability with 851 nm/s and 606 nm/s, respectively. The blood-brain partition coefficient is predicted by QPlogBB. All the compounds listed have shown the ability to cross the blood–brain barrier with values ranging from -0.05 to -1.62. All the best hits showed HOA value 3 indicating better oral absorption. PHOA ranges from 74 to 100, PSA ranges from 61.16 to 115.48 and QPlogKhsa ranges from -0.07 to 1.11. None of the compounds violated the rule of five and two compounds (1A and 1B) violated the rule of three (ROT).

**Table 4:** Prediction of ADME properties of ten hits. *PHOA*, percent human oral absorption; *ROF*, rule of five; *ROT*, rule of three; *HOA*, human oral absorption; *RV*, recommended values

Ligand	Mol. wt	donorHB	AccptHB	QPlogPo/w	QPlogS	QPPCaco	QPlogBB
1A	366.50	1	4.25	4.76	− 5.81	391.67	− 0.39
1B	380.53	1	4.25	4.98	− 5.79	389.63	− 0.36
1C	299.33	2	4.25	3.14	− 4.59	41.69	− 1.62
1D	299.33	2	4.25	3.14	− 4.58	43.15	− 1.60
1E	377.42	1	7.00	2.82	− 5.57	273.13	− 1.10
1F	362.35	1	5.50	3.79	− 5.67	851.00	− 0.65
1G	405.46	1	8.00	3.09	− 5.42	362.94	− 1.22
1H	419.48	2	9.00	2.67	− 4.72	357.04	− 1.11
1I	376.45	1	6.25	3.76	− 5.19	606.32	− 0.05
1 J	431.43	2	9.00	3.35	− 5.68	498.36	− 1.10
RV	130–725	0–6	2–20	− 2–6.5	− 6.5–0.5	< 25 poor; > 500 great	− 3–1.2
Ligand	HOA	PHOA	PSA	#Metab	QPlogKhsa	ROF	ROT
1A	3	100	61.16	5	1.02	0	1
1B	3	100	63.04	5	1.11	0	1
1C	3	74	104.22	2	0.06	0	0
1D	3	74	103.50	2	0.05	0	0
1E	3	87	103.89	3	0.24	0	0
1F	3	100	79.67	0	0.42	0	0
1G	3	91	115.48	2	0.04	0	0
1H	3	88	114.02	3	− 0.07	0	0
1I	3	100	62.86	4	0.62	0	0
1 J	3	94	113.12	3	0.13	0	0
RV	3-high	Max 100	Max 200	1–8	− 1.5–1.5	Max 4	Max 3

### Molecular dynamic simulation

Compound 1A with best XP dock score (-13.3) and compound 1I with best MM-GBSA score (-70.9) were exposed to molecular dynamic simulation for 40 ns. Overall, 1000 frames were generated in the trajectory. Protein-ligand interaction stability throughout the simulation was studied by RMSD (root mean square deviation) analysis.

**Fig. 4:**
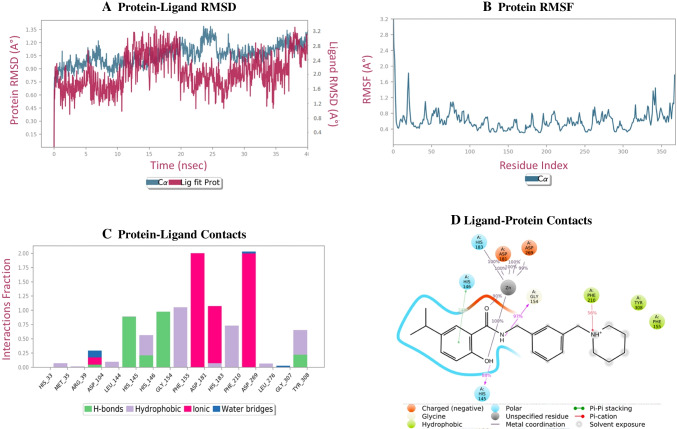
**A **Plot presenting the stability of protein-ligand interaction (RMSD). **B** The protein conformation changes along its side chain is represented in the RMSF throughout the trajectory. **C **and **D** represent bar graph and 2D interaction between ligand and protein throughout trajectory

Figure 4A demonstrates RMSD for 1A-HDAC 2 complex and was almost stable throughout the simulation. However, slight drift was observed at 2 to 11 ns, 21 to 28 ns and 35 to 37 ns. Figure 4B demonstrates the conformational changes taking place along the HDAC 2 protein side chain. RMSF (root mean square fluctuation) data of protein depicts the flexibility from 0.40 to 2.3 Å. Ligand-protein interactions were analysed throughout the simulation. XP docking protein-ligand interaction of compound 1A and MD simulation protein-ligand interaction were compared. It retained hydrogen bond interaction with GLY 154, hydrophobic interaction with PHE 155, PHE 210 and TYR 308, charged negative interaction with ASP 181 and ASP 269, polar interaction with HIS 145, HIS 146 and HIS 183 from XP docking. In addition, it also showed, H-bond interaction with HIS 154 and pi-pi stacking with HIS 146 during molecular dynamic simulation. Interactions of compound 1A-protein complex is shown in Fig. 4C and D.

**Fig. 5:**
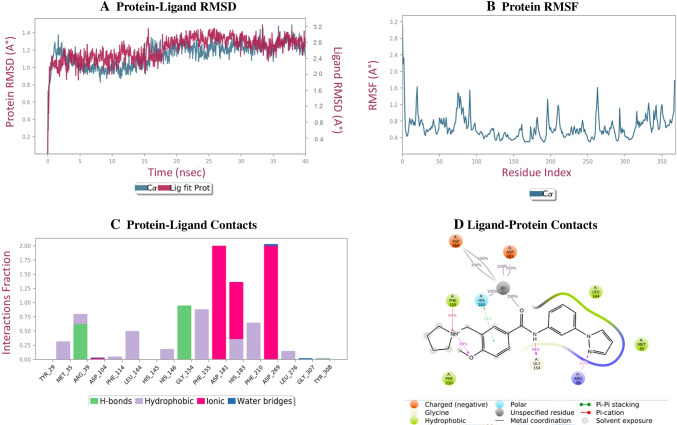
**A** Plot presenting the stability of protein-ligand interaction parameter used RMSD. **B** The protein conformation changes along its side chain is represented in the RMSF throughout the trajectory. **C** and **D** represents bar graph and 2D interaction between ligand and protein throughout trajectory

Figure 5A depicts the RMSD analysis of 1I - HDAC 2 complex which was stable throughout the simulation. Figure 5B demonstrates the conformational changes taking place in the HDAC 2 protein side chain. Root mean square fluctuation (RMSF) data of protein depicts the flexibility from 0.4 to 2.3 Å. XP docking protein-ligand interaction of compound 1I and MD simulation protein-ligand interaction were compared. The observation of interaction depicted that molecular dynamic simulation interaction have retained hydrophobic interaction with MET 35, LEU 144, PHE 155 and PHE 210, charged positive interaction with ARG 39; H-bond interaction with GLY 154, charged negative interaction with ASP 181 and ASP 269 and polar interaction with HIS 183 from XP docking. In addition, it also formed pi-pi stacking interaction HIS 183 and H-bond interaction with ARG 39 during molecular dynamic simulation. Interaction of compound 1I with HDAC 2 is shown in Fig. 5C and D.

Both the compounds exhibited stable protein–ligand complex throughout 40 ns simulation. Compound 1I has maximum MM-GBSA score and showed stable interaction with HDAC 2 protein. Even though it has lesser XP dock score than 1A, its dock score was more than the positive control. Also, dock score of 1I with other HDAC isoforms were less than seven indicating that it may be a best HDAC 2 isoform selective inhibitor among the selected compounds.

## Conclusions

In this study, we have identified ten best compounds as potent HDAC 2 inhibitors and among these compounds 1I can be more selective towards HDAC 2 compared to other HDAC isoforms. We designed an e-pharmacophore model using ligand co-crystallized with HDAC 2 protein (PDB ID: 4LY1) and compounds were subjected to e-pharmacophore-based virtual screening. Filtered compounds were subjected to structure-based virtual screening. Based on the docking scores, ten best hits were selected. All the hits showed better XP docking score with the minimum value of -12 and better than SAHA and MS-275. The best hits were subjected to virtual screening against other HDAC isoforms. Further, they were subjected to ADME and MM-GBSA score prediction. Based on docking score, ADME and MM-GBSA results, all the hits were efficient to be developed as potent HDAC 2 inhibitors. However, two best hits, one with top docking score and the other one with top MM-GBSA score were selected and subjected to molecular dynamic simulation. Molecular dynamic simulation of these compounds exhibited stable protein-ligand interaction throughout the simulation. Further, by validating the potency of selected lead molecules, this study could aid in developing selective HDAC 2 inhibitors.

## Supplementary Information

Below is the link to the electronic supplementary material.

Supplementary file1 (DOCX 536 KB)

## Data Availability

Not applicable.

## Abbreviations

ADME: Absorption, distribution, metabolism and excretion
HAT: Histone acetyltransferase
HDAC: Histone deacetylase
HDACIs: Histone deacetylase inhibitors
HTVS: High throughput virtual screening
MM-GBSA: Molecular mechanics-generalised born surface area
NCI: National Cancer Institute
NIH: National Institute of Health
PPW: Protein preparation wizard
RMSD: Root mean square deviation
RMSF: Root mean square fluctuation
SAHA: Suberoylanilide hydroxamic acid
SP: Standard precision
SPC: Simple point charge
XP: Extra precision
