# Supplementary material for: Identification of potent HDAC 2 inhibitors using E-pharmacophore modelling, structure-based virtual screening and molecular dynamic simulation
Source: J Mol Model. 2022 Apr 13;28(5):119. doi: 10.1007/s00894-022-05103-0 (PMC9007783; doi:10.1007/s00894-022-05103-0)
Supplement: Supplementary file 1 — Supplementary file1 (DOCX 536 KB) [file 894_2022_5103_MOESM1_ESM.docx]

**Supplementary information**

**Identification of potent HDAC 2 inhibitors using E-pharmacophore modeling, structure based virtual screening and molecular dynamic simulation**

Padmini Pai^1^, Avinash Kumar^2^, Manasa G. Shetty^1^, Suvarna Ganesh Kini^2^, Manoj Bhat Krishna^3^, Kapaettu Satyamoorthy^4^, Kampa Sundara. Babitha^1*^

^1^Department of Biophysics, Manipal School of Life Sciences, Manipal Academy of Higher Education, Manipal, Karnataka, India.

^2^Department of Pharmaceutical Chemistry, Manipal College of Pharmaceutical Sciences, Manipal Academy of Higher Education, Manipal, Karnataka, India.

^3^Department of Bioinformatics, Manipal School of Life Sciences, Manipal Academy of Higher Education, Manipal, Karnataka, India.

^4^Department of Cell and Molecular Biology, Manipal School of Life Sciences, Manipal Academy of Higher Education, Manipal, Karnataka, India.

^*^Corresponding author:

Dr. Kampa S. Babitha

Department of Biophysics

Manipal School of Life Sciences

Manipal Academy of Higher Education

Manipal, Karnataka – 576 104, India.

E-mail: babitha.ks@manipal.edu

We have redocked the ligand 4-(acetylamino)-N-[2-amino-5-(thiophen-2-yl)-phenyl]-benzamide with HDAC2 and XP dock score was found to be -13.05 kcal/mol. The RMSD value of the redocked ligand and the ligand in the X-Ray structure was found to be 0.25 Å [RMSD value below 1.5 is acceptable [1]. The superimposed structures of cocrystalised and redocked ligand is shown below.


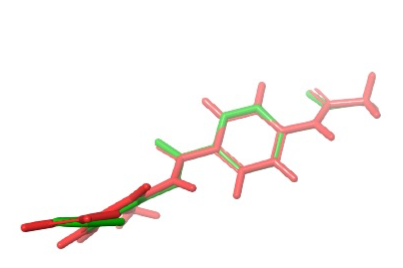


**Fig S1** Superimposed structure of co-crystallized ligand (Green) and redocked co-crystallized ligand (Red) with RMSD value - 0.25 Å.

2D interaction of the ligand with the HDAC2 enzyme is shown below.

| **a**  **b**    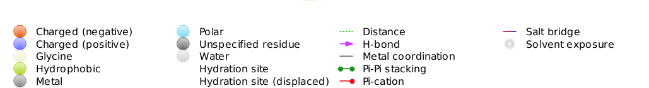 | 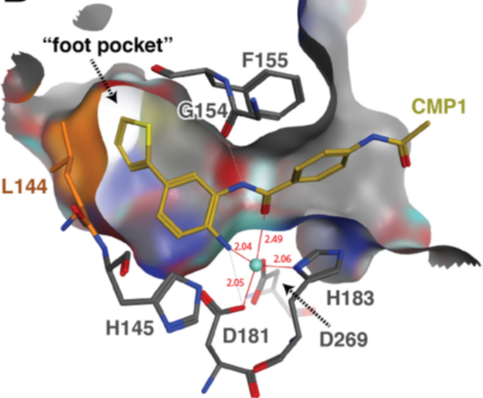 |
| --- | --- |

**Fig S2 (a)**2D Structure of redocked co-crystallized ligand with HDAC2 protein. **(b)** x-crystallography of ligand-protein interaction

RMSD value validates the docking study. 2D structure and the comparison of the interacted amino acids between X-ray crystallography and redocked ligand-protein complex further validates our study [2].

References:

1. Hevener KE, Zhao W, Ball DM, Babaoglu K, Qi J, White SW, and Lee RE (2009) Validation of molecular docking programs for virtual screening against dihydropteroate synthase. J Chem Inf Model 49:444-460. Doi:10.1021/ci800293n
2. Lauffer BE, Mintzer R, Fong R, Mukund S, Tam C, Zilberleyb I and Ortwine DF (2013). Histone deacetylase (HDAC) inhibitor kinetic rate constants correlate with cellular histone acetylation but not transcription and cell viability. J Biol Chem 288:26926-26943. Doi:10.1074/jbc.M113.490706
